# Altered Inter-Subregion Connectivity of the Default Mode Network in Relapsing Remitting Multiple Sclerosis: A Functional and Structural Connectivity Study

**DOI:** 10.1371/journal.pone.0101198

**Published:** 2014-07-07

**Authors:** Fuqing Zhou, Ying Zhuang, Honghan Gong, Bo Wang, Xing Wang, Qi Chen, Lin Wu, Hui Wan

**Affiliations:** 1 Department of Radiology, The First Affiliated Hospital, Nanchang University, Nanchang, Jiangxi Province, China; 2 Jiangxi Province Medical Imaging Research Institute, Nanchang, Jiangxi Province, China; 3 Department of Oncology, The Second Hospital of Nanchang, Nanchang, Jiangxi Province, China; 4 Department of Neurology, The First Affiliated Hospital, Nanchang University, Nanchang, Jiangxi Province, China; Tianjin Medical University General Hospital, China

## Abstract

**Background and Purpose:**

Little is known about the interactions between the default mode network (DMN) subregions in relapsing-remitting multiple sclerosis (RRMS). This study used diffusion tensor imaging (DTI) and resting-state functional MRI (rs-fMRI) to examine alterations of long white matter tracts in paired DMN subregions and their functional connectivity in RRMS patients.

**Methods:**

Twenty-four RRMS patients and 24 healthy subjects participated in this study. The fiber connections derived from DTI tractography and the temporal correlation coefficient derived from rs-fMRI were combined to examine the inter-subregion structural-functional connectivity (SC-FC) within the DMN and its correlations with clinical markers.

**Results:**

Compared with healthy subjects, the RRMS patients showed the following: 1) significantly decreased SC and increased FC in the pair-wise subregions; 2) two significant correlations in SC-FC coupling patterns, including the positive correlation between slightly increased FC value and long white matter tract damage in the PCC/PCUN-MPFC connection, and the negative correlations between significantly increased FC values and long white matter tract damage in the PCC/PCUN-bilateral mTL connections; 3) SC alterations [log(N track) of the PCC/PCUN-left IPL, RD value of the MPFC-left IPL, FA value of the PCC/PCUN-left mTL connections] correlated with EDSS, increases in the RD value of MPFC-left IPL connection was positively correlated to the MFIS; and decreases in the FA value of PCC/PCUN-right IPL connection was negatively correlated with the PASAT; 4) decreased SC (FA value of the MPFC-left IPL, track volume of the PCC/PCUN-MPFC, and log(N track) of PCC/PCUN-left mTL connections) was positively correlated with brain atrophy.

**Conclusions:**

In the connections of paired DMN subregions, we observed decreased SC and increased FC in RRMS patients. The relationship between MS-related structural abnormalities and clinical markers suggests that the disruption of this long-distance “inter-subregion” connectivity (white matter) may significantly impact the integrity of the network's function.

## Introduction

Pathological [1]–[4] and neuroimaging [5]–[7] studies have demonstrated the widespread involvement of the gray matter and white matter of the brain in multiple sclerosis (MS). In MS, minimal initial structural damage can be sufficient to trigger a strong response in function and hyperactivity [8], [9]. Structural damage limits functional reorganization [10] until burnout, resulting in hypoactivity and dysfunction [11]. Although the structures has been considered as the underlying foundation of functional change. But in fact, it is controversial in the relationship between the cerebral structure and function or clinical function [12]–[14]. Understanding the pathophysiological mechanisms relating structural damage and functional alterations in MS and its correlations with clinical indices remains a challenge in clinical research [15]–[17].

Recent studies of MS have reported significant synchronization or connectivity alterations in diverse disease stages [8], [9], [18], [19], including changes in the default-mode network (DMN) [11], [18]–[20], sensorimotor network (SMN) [21], [22] and frontoparietal network (FPN) [9], [23] (reviewed in [24]). The DMN, a recent focus of functional MRI studies of MS, may be particularly important for understanding the impact of the disease on the brain [25], [26]. Especially in relapsing-remitting MS (RRMS), different alterations within the DMN have been described, including decreased [27], increased [19] and mixed [18] changes as well as no significant differences [8]. Widespread functional abnormalities between the DMN and other large-scale networks [28] have also been reported, but the correlation between significantly altered brain activity and clinical characteristics/neuropsychological tests scores is inconsistent [24].

The DMN comprises multiple dissociated subregions that are engaged to facilitate functional construction related to cognition through the long-distance connectivity “backbone”. In contrast to local interactions, this long-distance cortico-cortical (inter-subregion) interaction is involved in the global communication of the whole network [29]. The anatomical and functional connectivity (FC) linking to paired DMN subregions has been accessed using DTI tractography and resting-state functional connectivity [13], [14], [30]–[33].

However, little is known about the connectivity “backbone” linking the diverse DMN subregions in MS. Studying the long-distance (“backbone”) inter-subregion connections may provide more information that is critical to fully understanding MS-related functional alterations. In this study, we hypothesized that both structural connectivity (SC) and FC in paired DMN subregions are disrupted, as are its correlations with clinical indices in RRMS. To test this hypothesis, we combined probabilistic tractography and pair-wise FC to characterize the connectivity of long white matter tracts and the synchrony of intrinsic neuronal activity in paired DMN subregions and to examine the mechanisms that might underlie the observed alterations. Studying the relationship between the SC and FC of anatomically separate DMN subregions may enrich our understanding of the neural underpinnings of RRMS.

## Materials and Methods

### Participants

We recruited 24 patients with clinically definite MS at the First Affiliated Hospital of Nanchang University from May 2010 to December 2013, according to McDonald's criteria [34]. The inclusion criteria for the patients were as follows: RRMS course [35]; Expanded Disability Status Scale (EDSS) score <2.5 (corresponding to minimally disabled [36]); and treatment with immunomodulatory medication (20 with β-interferons, 4 with Glatiramer acetate). None of the recruited patients had any relapses or cortico-steroid treatment during the month preceding the MR acquisition. Twenty-four healthy control participants from the local community were matched to the patients individually for sex, age, and education level. Their clinical and demographic data are shown in Table 1. The present study was approved by the Medical Research Ethics Committee and the Institutional Review Board of the First Affiliated Hospital of Nanchang University. All subjects signed written consent forms for participation in the study.

**Table 1:** Demographics and clinical characteristics of the control subjects and RRMS patients.

	RRMS patients (n = 24)	Control subject (n = 24)	P values
Gender (M/F)	8/16	8/16	>0.99
Mean age (range) (years)	39.5 (20–56)	39.6 (21–56)	0.99
Mean disease duration (range) (months)	34.1 (3–187)	-	-
Mean TWMLL* (range) (ml) (normalized)	18.17 (0.43–79.41)	-	-
Mean BPF (range)	0.828 (0.78–0.86)	0.853(0.82–0.88)	0.000
Median EDSS (range)	1.604 (1.0–2.5)	0	-
Mean PASAT (range)	84.0 (61–103)	97.87 (82–118)	0.002
Mean MFIS-5 (range)	11.29 (6–17)	0.29 (0–1)	0.000

Note: M = male; F = female; TWMLL = total white matter lesion loads; BPF = brain parenchymal fraction; EDSS = expanded disability status scale; PASAT = paced auditory serial addition test; MFIS = modified fatigue impact scale;

*The measurement procedures for TWMLL and BPF in the RRMS patients have been previously described by [37] and [38], respectively (see Figure S1 and Supporting Information S1).

### Image acquisition

All participants were scanned using a 3.0 T MRI scanner (Trio Tim, Siemens Medical Systems, Erlangen, Germany). *T*_2_ *W*, *T*_1_ *W*, DTI and resting-state fMRI (rs-fMRI) images were acquired using the following sequences: 1) *T*_2_-weighted turbo spin echo imaging (repetition time [TR]/echo time [TE] = 5100/117 ms; number of excitations [NEX] = 3; echo train length = 11; matrix = 416×416; field of view [FOV] = 240×240 mm; slice number = 22; slice thickness = 6.5 mm; and orientation = axial); 2) *T*_1_-weighted 3-D imaging (TR/TE = 1900/2.26 ms; NEX = 1; matrix = 240×256; FOV = 215×230 mm; slices number = 176; slice thickness = 1.0 mm; and orientation = sagittal); 3) spin echo single-shot echo planar imaging (TR/TE = 7200/104 ms; NEX = 2; matrix = 128×128; FOV = 230×230 mm; slices number = 49; slice thickness = 2.5 mm; orientation = axial; 64 nonlinear diffusion weighting gradient directions with *b* = 1000 s/mm^2^ and 1 additional image without diffusion weighting [i.e., b = 0 s/mm^2^]); and 4) rs-fMRI scan using an echo planar imaging (EPI) sequence with the following parameters: TR/TE = 2000/30 ms; flip angle = 90°; FOV = 200×200 mm; matrix = 64×64; 30 interleaved axial slices with 4-mm thickness with an interslice gap of 1.2 mm; and number of time points = 240. During rs-fMRI scanning, subjects were instructed to keep their eyes closed, not to think about anything in particular, and not to fall asleep. A foam pad was used to minimize the head motion of all subjects.

### Functional MRI data preprocessing

The rs-fMRI data were preprocessed using the SPM8 software package (http://www.fil.ion.ucl.ac.uk) and the Data Processing Assistant for Resting-State fMRI Advanced Edition (DPARSFA) V2.2 (http://www.restfmri.net) running in Matlab 2012a (Mathworks, Natick, MA, USA). This process involved removing the first 10 time points, slice timing, voxel-specific head motion calculation and correction to adjust the time series of the images, using unified segmentation of the *T_1_*-weighted images for non-linear registration and normalization of the functional images to the Montreal Neurological Institute (MNI) space with 3×3×3 mm^3^ re-sampling, then spatial smoothing was performed using a 6-mm full–width-half-maximum Gaussian kernel. We used temporal band-pass filtering (0.01 Hz<f<0.08 Hz) and multiple regression analysis of nuisance variables from the BOLD data, which included a ventricular signal averaged from ventricular regions of interest (ROIs), a white matter signal averaged from white matter ROIs, a whole brain signal averaged across the whole brain, six head realignment parameters obtained by rigid body head motion correction, the derivatives of each of these signals and a voxel-specific head motion parameter [39].

### Default mode network subregion extraction

**Figure 1:**
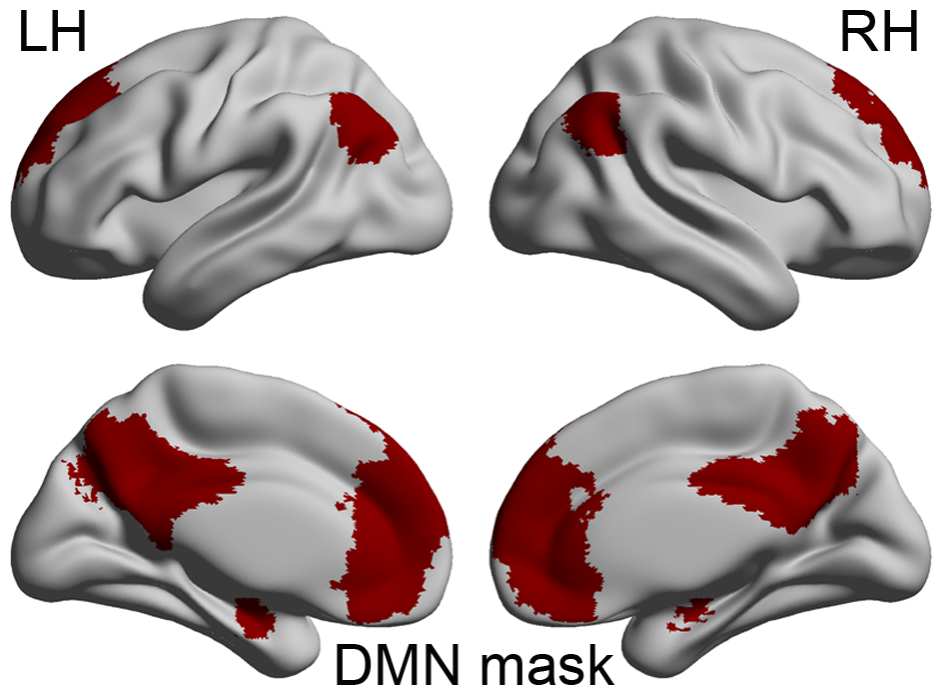
The union of the DMN mask identified and extracted with independent component analysis from RRMS and healthy control groups (LH: left hemisphere; RH: right hemisphere).

The default mode network subregions were extracted using a similar method to that described in a previous study [14], which is briefly described as follows:

1. Group independent component analysis (ICA) was first performed using the Group ICA of fMRI Toolbox (GIFT) (http://icatb.sourceforge.net/, vision 2.0e) with the Infomax algorithm, which decomposed the smoothed data of each individual in the RRMS and healthy control groups into 63 and 69 independent components (estimated by the minimum description length (MDL) criterion [40]), respectively. The RRMS and healthy control groups in this study were estimated and analyzed separately to prevent the mixing of the specific resting-state network pattern from each group [41].
2. The largest spatial correlation with DMN templates was used to select the components to be retained for further analysis. The DMN templates form the Medical Image Analysis (MIA) laboratory (http://mialab.mrn.org/index.html), which was divided into six subregions: bilateral inferior parietal lobules (IPLs), bilateral medial temporal lobes (mTLs), posterior cingulate cortex/precuneus (PCC/PCUN) and medial prefrontal cortex (MPFC). Each of the subregions was manually divided into ROIs based on the union of the DMN maps from the RRMS and healthy control groups (REST V1.8, http://www.restfmri.net). Detailed information on ROIs is shown in Figure 1 and Table S1.
3. These six subregions were used for subsequent SC and FC analyses in the patient and control groups, respectively.

### Calculation of synchrony in the intrinsic neuronal activity of pair-wise subregions

**Figure 2:**
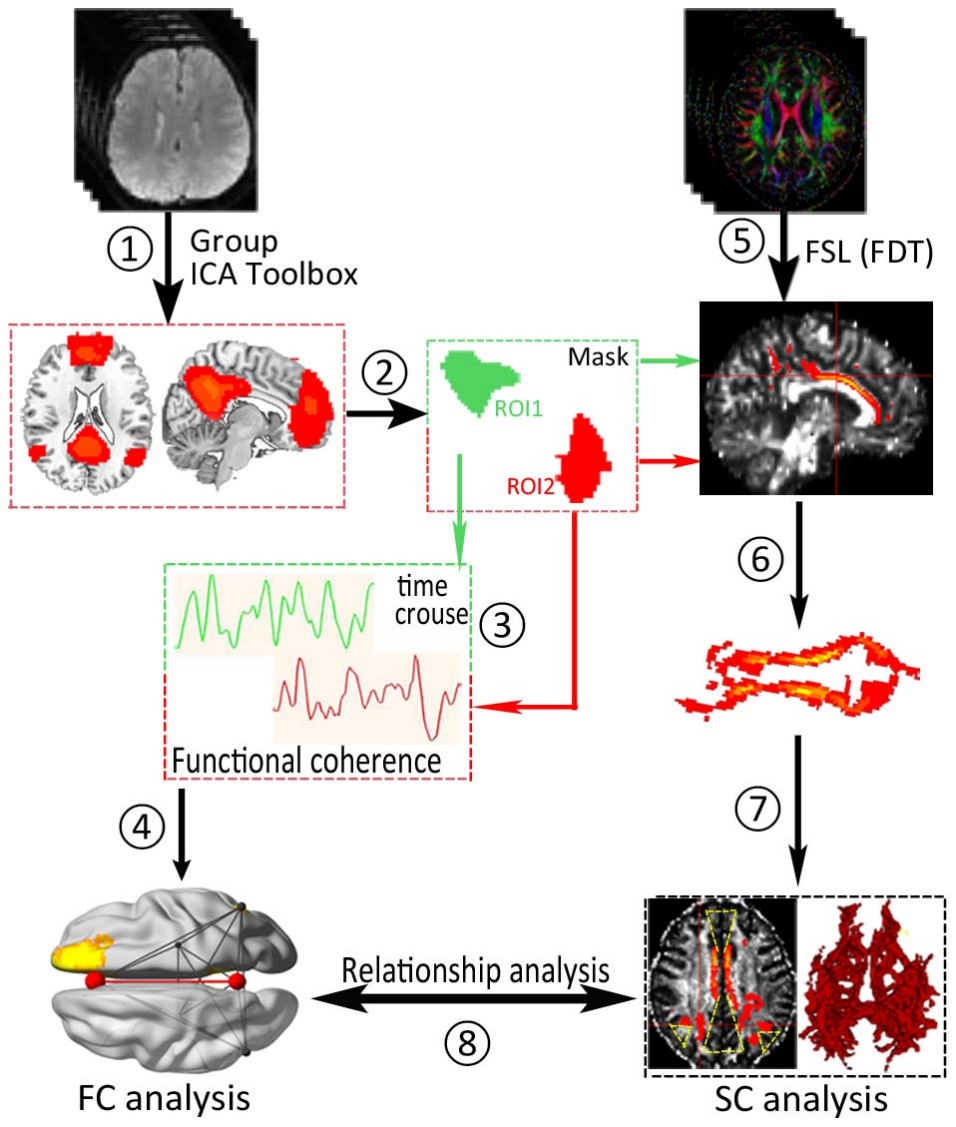
A flowchart of calculating the correlation between structural connectivity and functional connectivity within the default mode network (DMN) subregions. (1) DMN extraction was performed by ICA. (2) The subregions were extracted as regions of interest (ROIs) within the DMN. (3) The time series in each ROI was extracted with the DMN. (4) The temporal correlation coefficients between each ROI pair were quantified within the DMN. (5) Preprocessed motion and eddy current distortion correction of the model distributions of the relevant parameter (Monte Carlo sampling) was performed using FMRIB's diffusion toolbox (FDT v2.0). (6) Pair-wise ROI (AND logic) probabilistic tractography was used to calculated the distribution of fiber orientations. (7) Features of the long white matter tracts (above threshold 0.2) remaining fiber bundles connecting each pair of ROIs were compared between the two groups, including the strength of a pathway and the above-threshold standard DTI parameters.

To extract and compute the time series of each pair of subregions from two groups, we used a similar method to that described in previous studies [32], [41], including multiple regression of nuisance variables and band-pass filtering (0.01–0.08 Hz). Temporal correlation coefficients were calculated only between each pair of regions using the *Pearson* correlation to quantify the FC values (Figure 2).

### Characterizing the connectivity of long white matter tract from DTI data

To investigate the SC of pair-wise subregions, the group-space ROI was coregistered to individually (native) DTI space using FLIRT methods [42]. They are briefly described as follows:

1. Using FMRIB's diffusion toolbox (FDT v2.0, http://fsl.fmrib.ox.ac.uk/fsl/fslwiki/FDT), DTIFIT was used to fit a single tensor model at each voxel of the preprocessed eddy current corrected diffusion weighted data.
2. Probabilistic tractography was performed using FMRIB's diffusion toolbox (FDT v2.0, http://fsl.fmrib.ox.ac.uk/fsl/fslwiki/FDT). BEDPOSTX was used to model 5000 iterations within each voxel with a curvature threshold of 0.2, a step length of 0.5 and a maximum number of 2000 steps [43]. Pair-wise ROIs (“AND” logic) were used to calculate a distribution of fiber orientations.
3. The connectivity of long white matter tracts was at a normalized probability value of 0.20 [31], [44] and visually inspected to confirm successful tracing in each individual by multiple experienced technicians.
4. Features of the long white matter tracts (above threshold) connecting each pair of ROIs were compared between the two groups. The features included the strength of a pathway, indicated by the volumes and mean track count per WM region of the paired ROIs [43], and the above threshold standard DTI parameters, including fractional anisotropy (FA) and three diffusivity measurements [average mean diffusivity (MD), axial diffusivity (AD) and radial diffusivity (RD)].

### Comparison of structure and function

We assessed the character of the SC and FC measures in three steps: 1) structural and functional connections were identified of pair-wise DMN subregions, specifically, the fiber connections as being either present or absent, identified by visual inspection; 2) the connectivity strength of pair-wise subregions was determined and inter-groups compared separately for FC and SC; and 3) the relationship of SC-FC coupling was determined by linear correlation analysis for each pair-wise connection. Pair-wise probabilistic tractography tracks in which a significant difference was detected between groups were excluded from this analysis.

### Statistical analysis

For the FC and SC separately, we compared the 15 pairs FC between the ROIs (i.e., PCC/PCUN-MPFC, MPFC-right IPL) and eight pairs SC using repeated one-way repeated-measures analysis of variance (ANOVA) (SPSS Inc., Chicago, IL, USA). Post-hoc T-tests were used to identify differences in SC and FC between two groups (*P*<0.05, Bonferroni corrected). Linear regression analysis was performed using SPSS 13.0 (SPSS Inc., Chicago, IL, USA) to investigate the relationship between neuropsychological tests scores and damage to SC/FC and the effect on clinical parameters and indices within RRMS patients. The threshold was set at a significance level of *P*<0.05 and was corrected for multiple comparisons using the Bonferroni correction.

## Results

### Default mode network subregion extraction by ICA analysis

In the present study, DMN maps revealed a typical spatial pattern in both the patient and control groups (*P*<0.05, FDR corrected), and six subregions were extracted from the union of the DMN maps of two groups (Figure 1). The details of the brain regions in the DMN subregions are reported in the supporting materials (Table S1).

### Functional connectivity measures of pair-wise subregion coherence

**Figure 3:**
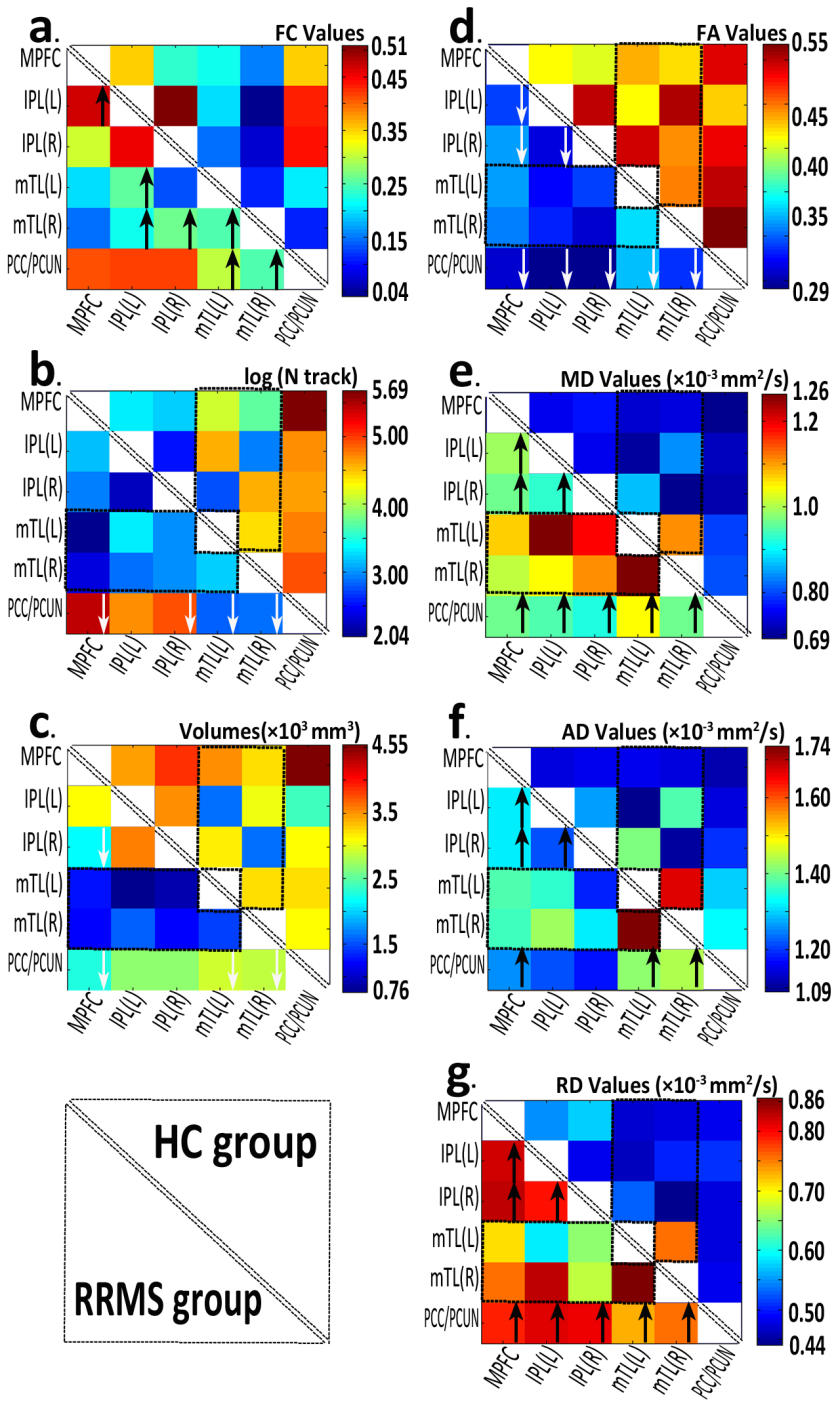
A set of matrices of functional and structural analyses of pair-wise default mode network subregions in the RRMS patient and healthy control (HC) groups. The figure (a) is a correlation matrix of average time series of subregions (ROIs) in the network (subregion labels are on the left and bottom). The figures (b–s) show the strength of the structural connectivity matrix [of average log (N track) and volumes] of each pair-wise connection. The figures (d–g) are above-threshold (0.2) standard DTI parameters: including the fractional anisotropy (FA), mean diffusivity (MD), axial diffusivity (AD) and radial diffusivity (RD) of each pair-wise connection. The black arrow indicates increased and the white arrow decreased alteration in the patients with RRMS. Notably, structural connectivity was only compared without the dashed boxes.

Seed-based FC was then used to measure the FC strength for each pair-wise connection between the DMN subregions. Figure 3a presents the mean FC coefficients of the pair-wise subregions. An analysis of the FC coefficients of seven paired subregions (MPFC-left IPL, PCC/PCUN-left mTL, PCC/PCUN-right mTL, left IPL-left mTL, left IPL-right mTL, right IPL-right mTL, and left mTL-right mTL) showed significant differences between the two groups at *P*<0.05 (ANOVA; Tables S2 and S3).

### Structural connectivity measures of long white matter tracts between pair-wise subregions

**Figure 4:**
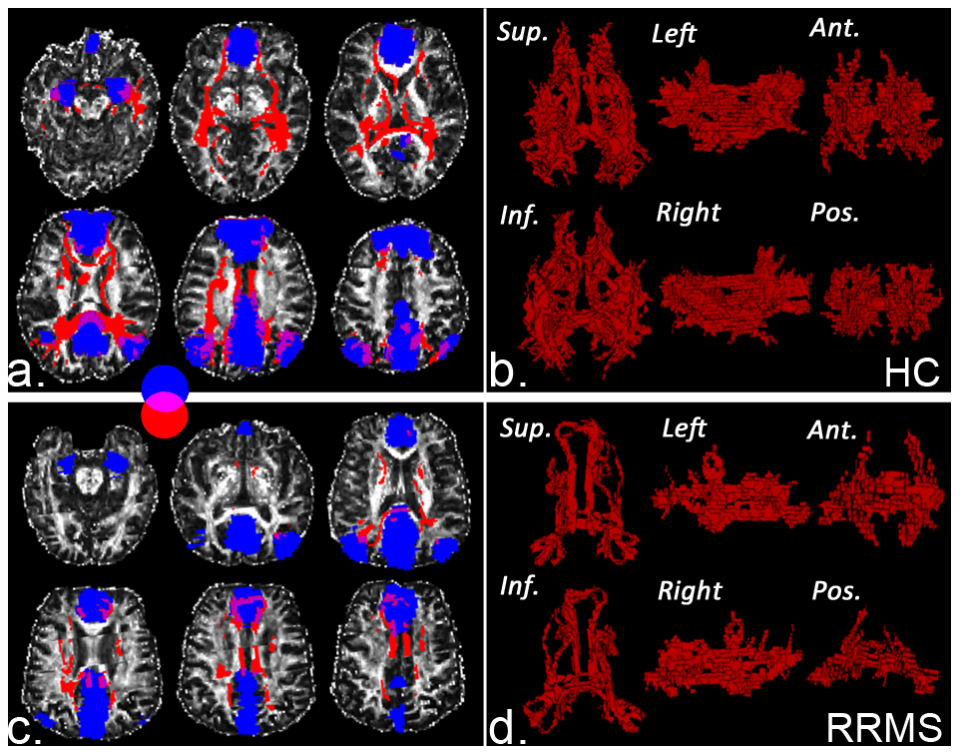
An example of the long white matter fibers of pair-wise subregions within the default mode network detected by probabilistic tractography in one control subject (a) and one RRMS patient (c) in the native space. Figures (b) and (d) correspond to 3-dimensional probabilistic tractography. (red: fiber tract, blue: mask of subregions, Sup = Superior, Inf = Inferior, Ant = Anterior, Pos = Posterior).

Figure 4 showes the long white matter tracks identified by probabilistic tracking between pair-wise subregions. Compared with the healthy control group, a lower probabilistic track number was detected in the RRMS group (Table S3 and examples in Figure S2).

In this study, we only compared the structural connectivity measures of non-significant differences in detectable tractography (eight connections) in the pair-wise subregions between two groups. The SC strength was assessed by measuring the volumes and mean track count along the above-threshold reconstructed tracks (Figures 3b, 3c and Tables S2, S3). In the patients with RRMS, smaller volumes were detected in the track linking the PCC/PCUN and the MPFC (4.55×10^3^ mm^3^ vs. 2.26×10^3^ mm^3^) and the track linking the MPFC and the right IPL (3.88×10^3^ mm^3^ vs. 2.17×10^3^ mm^3^); lower counts [log(N track)] were detected in the track linking the PCC/PCUN and the MPFC (5.69 vs. 5.21), the track linking the PCC/PCUN and the left mTL (4.77 vs. 2.83), and the track linking the PCC/PCUN and the right mTL (4.90 vs. 2.84).

The integrity of the above-threshold reconstructed tracks was assessed by measuring the standard DTI parameters (Figures 3d–3g and Tables S2, S3). In the patients with RRMS, decreased FA and increased diffusivity measurement (MD, AD and RD) values were detected in all eight tracts (*P*<0.05, Table S3), with the exception of the AD values of the track linking the PCC/PCUN and the bilateral IPL, and the track linking between the bilateral IPLs.

### Relationship between structure and function measures

**Figure 5:**
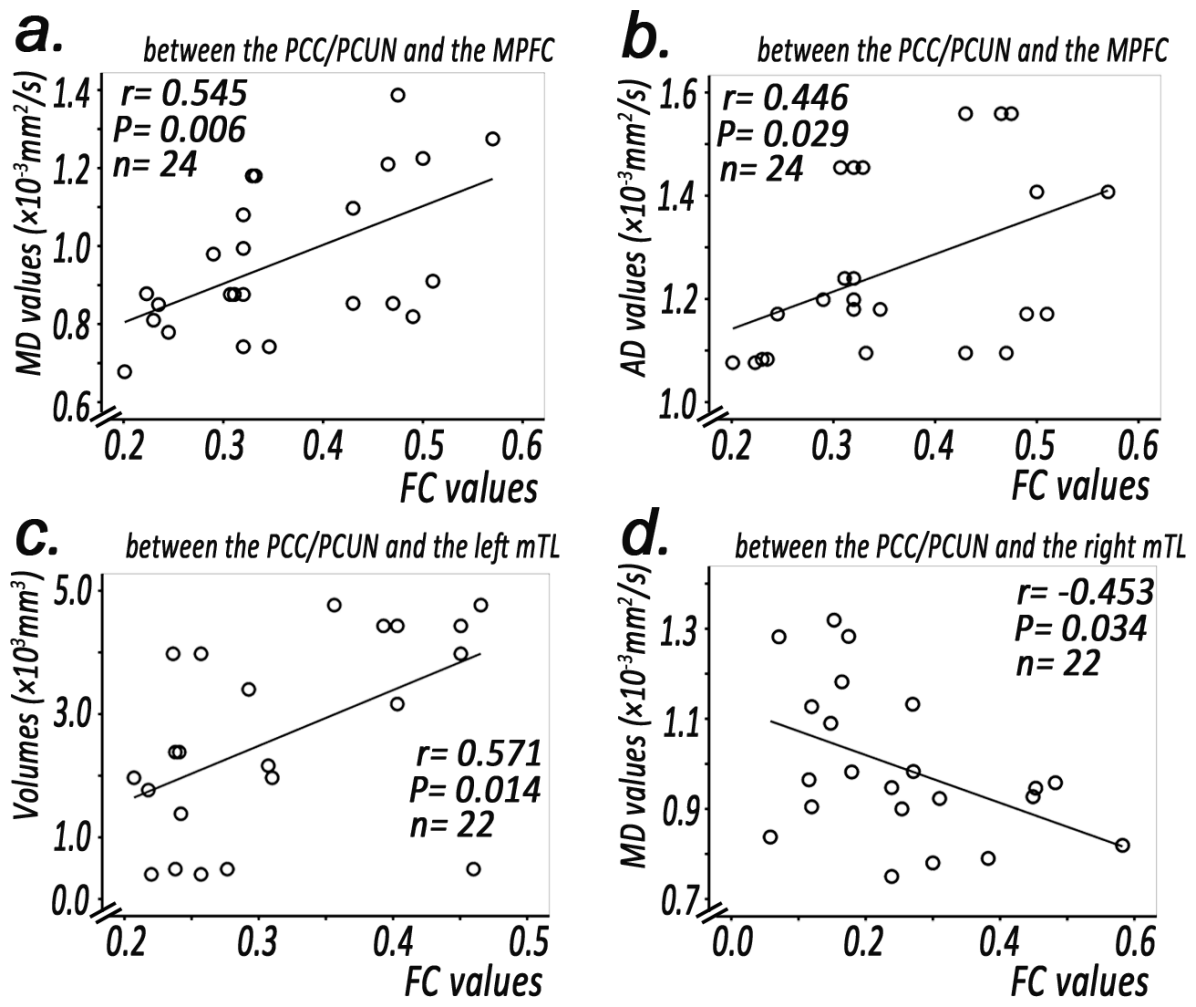
The Pearson correlation between structural connectivity (SC) indices (y axis) and functional connectivity (FC) indices (x axis) of pair-wise subregions within the DMN of RRMS patients. The FC value positively correlated with the MD value (a) and FC value (b) of the tract between the PCC/PCUN and the MPFC in RRMS patients (P<0.05, Bonferroni corrected). The FC values was positively correlated with the volume (c) of the tract between the PCC/PCUN and the left mTL in RRMS patients (P<0.05, Bonferroni corrected). The FC value positively correlated with the MD value (d) of the tract between the PCC/PCUN and right mTL (P<0.05, Bonferroni corrected). AD = axial diffusivity, IPL = inferior parietal lobule, MD = mean diffusivity, mTL = medial temporal lobes, MPFC = medial prefrontal cortex, PASAT = paced auditory serial addition test, PCC/PCUN = posterior cingulate cortex/precuneus, RRMS = relapsing–remitting multiple sclerosis.

Linear regression analyses (Figure 5) revealed that in RRMS patients, increases in the MD and AD values of the track linking the PCC/PCUN and the MPFC were positively correlated with slight increases in FC values (Figure 5a, *r* = 0.545, *P* = 0.006; Figure 5b, *r* = 0.446, *P* = 0.029, respectively). The smaller decrease in the volumes of the track linking the PCC/PCUN and the left mTL was positively correlated with increased FC values (Figure 5c, *r* = 0.517, *P* = 0.014). An increase in the MD values of the track linking the PCC/PCUN and the right mTL was negatively correlated with an increase in the FC value (Figure 5d, *r* = −0.453, *P* = 0.034). Figure S3 shows an example of the relationship between the FC and SC measures in others threshold (0.05, 0.10, 0.15, 0.20, 0.25, and 0.30) of probabilistic tractography.

### Linear regression results between abnormal connectivity indices and clinical markers of RRMS

**Figure 6:**
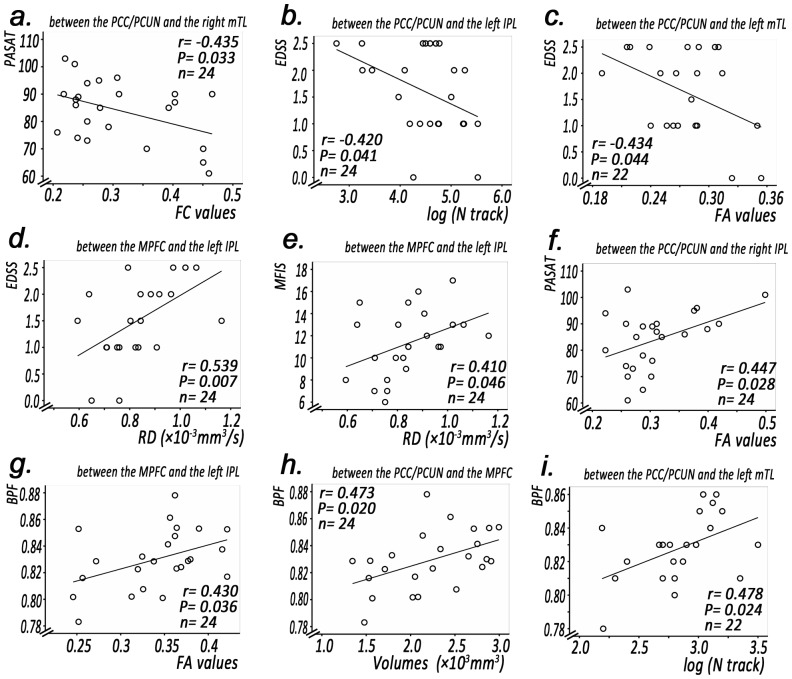
The Pearson correlation between structural/functional connectivity indices (x axis) and clinical markers (y axis) of pair-wise subregions within the DMN of RRMS patients. The functional connectivity (FC) value (a) between the PCC/PCUN and the right mTL negatively correlated with the PASAT in RRMS patients (P<0.05, Bonferroni corrected). The EDSS score negatively correlated with the log (N track) of the tract between the PCC/PCUN and the left IPL (b) and negatively correlated with the FA value of the tract between the PCC/PCUN and the left mTL (c) in RRMS patients (P<0.05, Bonferroni corrected). The EDSS score positively correlated with the MD value of the tract between the MPFC and the left IPL (d) in RRMS patients (P<0.05, Bonferroni corrected). The PASAT score positively correlated with the FA value of the tract between the PCC/PCUN and the right IPL (e) in RRMS patients (P<0.05, Bonferroni corrected). The MFIS score negatively correlated with the MD values of the tract between the MPFC and the left IPL (f) in RRMS patients (P<0.05, Bonferroni corrected). The BPF score positively correlated with the FA value of the tract between the MPFC and the left IPL (g) in RRMS patients (P<0.05, Bonferroni corrected). The BPF score positively correlated with the volume of the tract between the PCC/PCUN and the MPFC (h) in RRMS patients (P<0.05, Bonferroni corrected). The BPF score positively correlated with the log (N track) value between the PCC/PCUN and the left mTL (i) in RRMS patients (P<0.05, Bonferroni corrected). AD = axial diffusivity, EDSS = expanded disability status scale, FA = fractional anisotropy, IPL = inferior parietal lobule, MD = mean diffusivity, mTL = medial temporal lobes, MPFC = medial prefrontal cortex, PASAT = paced auditory serial addition test, PCC/PCUN = posterior cingulate cortex/precuneus, RRMS = relapsing–remitting multiple sclerosis, TWMLL = total white matter lesion loads.

Linear regression analyses revealed that in RRMS patients, increases in FC values between the PCC/PCUN and left mTL were negatively correlated with the PASAT score (Figure 6a, *r* = −0.435, *P* = 0.033).

The mean track count of the track linking the PCC/PCUN and the left IPL was negatively correlated with the EDSS value (Figure 6b, *r* = −0.420 *P* = 0.041). A decrease in the FA value of the track linking the PCC/PCUN and the left mTL was negatively correlated with the EDSS value (Figure 6c, *r* = −0.434, *P* = 0.044). An increase in the RD value of the track linking the MPFC and the left IPL was positively correlated with the EDSS and MFIS values (Figure 6d, *r* = 0.539 *P* = 0.007; Figure 6e, *r* = 0.410, *P* = 0.046, respectively). A decrease in the FA value of the track linking the PCC/PCUN and the right IPL was positively correlated with the PASAT value (Figure 6f, *r* = 0.447, *P* = 0.028).

A decrease in the FA value of the track linking the MPFC and the left IPL was positively correlated with the BPF value (Figure 6g, *r* = 0.430 *P* = 0.036). A decreased volume of the track linking the PCC/PCUN and the MPFC was positively correlated with the BPF value (Figure 6h, *r* = 0.473 *P* = 0.020). The mean track count of the track linking the PCC/PCUN and the left mTL was positively correlated with the BPF value (Figure 6i, *r* = 0.478 *P* = 0.024).

## Discussion

In the present study, we showed a significantly decreased inter-subregion SC in the DMN and increased FC of pair-wise subregions in minimally disabled RRMS patients. We also found two significant correlations between the FC and SC measures: slightly increased FC value was positively correlated with long white matter tract damage in the PCC/PCUN-MPFC connection, and significantly increased FC values were negatively correlated with long white matter tract damage in the PCC/PCUN-bilateral mTL connections. Relationships between MS-related structural abnormalities and clinical markers were detected more often than relationships between functional abnormalities and clinical markers. This study, linking the increased inter-subregion functional coherence and decreased long white matter connections of DMN subregions, provides new insights into the functional reorganization in RRMS.

### Decreased connectivity of long white matter tracts between the default mode network subregions

DTI measures (region of interest analysis [6], [45] and tract-based spatial statistics [46], for example) are sensitive to white matter damage in MS, including interior focal lesions and normal appearing white matter, and these regional DTI alterations are related to disability and cognitive deficits [47]. Although damage to the integrity of fiber tracts has been investigated in MS, very little is known about alterations in the fiber connections between the distant subregions within the DMN. The long white matter tracts between subregions are the structural highways of the DMN, and they enable information to travel quickly from one subregion to another subregion to facilitate the ongoing interregional neuronal communication [14]. In contrast to deterministic tractography (just a single trajectory reconstruction), probabilistic tractography was performed in our study to investigate the strength of SC and the above-threshold standard DTI parameters in the long white matter tracts. Probabilistic tractography methods are used to propagate a large number of pathways passing through the seed point, and the pathway orientations are drawn from a distribution of possible orientations [43], [44]. The probabilistic tractography methods can be used to (facilitate) easily reconstruct the existence of direct neuroanatomical connections between DMN subregions [14], [30].

The potential of probabilistic tractography to detect abnormalities in the long white matter tracts of patients with MS has been suggested in recent studies [48]–[51]. These studies have reported the sensitive detection of tissue damage associated with cervical cord relapse [48] and damage to the corticospinal tract [49]–[51] and the transcallosal hand motor fibers [49], suggesting axonal degeneration and myelin breakdown in probabilistic white matter tracts, these measures correlate with disability [50], [51]. In our study, probabilistic tractography analysis showed decreases in SC strength and structural damage derived from standard DTI parameters (decreased FA and increases in three diffusivity measurements) in the tracts connecting the DMN subregions. These findings further support the existence of inter-subregion anatomical disconnection and microstructural damage (axonal and myelin loss) of entire long white matter tracts in the DMN [19].

Notably, with significant anatomical disconnection (without white matter connection) in the DMN of patients with RRMS, the SC strength and the derived standard DTI parameters were statistically compared only for connections within eight paired subregions showing no significant indifferences.

### Increased functional connectivity in the pair-wise default mode network subregions

In the present study, increased functional connectivity was observed in the patients with RRMS using pair-wise subregion coherence analysis, although not for all paired connections. Increased functional connectivity was detected in seven paired subregions (MPFC-left IPL, PCC/PCUN-left mTL, PCC/PCUN-right mTL, left IPL-left mTL, left IPL-right mTL, right IPL-right mTL, and left mTL-right mTL) within the DMN. Pair-wise base coherence analysis provides a simply and/or straightforward FC metric [52]. Recently, various MS-related FC alteration patterns have been reported using rs-fMRI, including decreased [27], increased [19] and mixed [18] changes as well as no significant differences [8]. Our results may suggest an increased neural effort in cortical activity due to the presence of structural damage the connecting pathways in minimally disabled RRMS patients [19].

### Relationship of SC-FC coupling measures

Functional reorganization might be an important pathophysiological factor in controlling information processing deficiencies in relatively early MS. Neuronal fiber tracts are bundles that have an extremely large number of axons that act as a bridge connecting two larger subregions over long distances [53], known as the connectivity “backbone”. In general, inter-subregions that are clearly and directly linked by anatomical long white matter tracts should exhibit a high level of functional communication [13]. The relationship pattern has previously investigated the use of both SC and FC analyses, which have suggested that the functionally linked DMN subregions reflect the underlying structural connectivity architecture [13], [14]. These findings have enriched our understanding of the neuro-pathophysiological mechanisms of disease [32], [41].

In this study, significant relationships between SC-FC coupling measures were observed in the connection among the PCC/PCUN and the MPFC (MD values vs. FC coefficients, *r* = 0.545, *P* = 0.006; AD values vs. FC coefficients, *r* = 0.446, *P* = 0.029, respectively), the connection between the PCC/PCUN and left mTL (volumes of the fiber tract vs. FC coefficients, *r* = 0.571, *P* = 0.014) and the connection between the PCC/PCUN and right mTL (MD values vs. FC coefficients, *r* = −0.453, *P* = 0.034). We identified three different MS-related relationships in SC-FC coupling measures: a) a slightly increased FC was positively correlated with damage to SC; b) significantly increased FC was negatively correlated with damage to SC; and c) dissociation of SC-FC coupling measures. The probabilistic tractography and resting-state functional connectivity approach is advantageous for characterizing the relationship of SC-FC coupling [31], [54]. In minimally disabled MS patients, increased synchronization in the DMN have been observed [8], revealing a compensatory mechanism in response to structural damage (relationship pattern a), which seems to be finite. At some point, structural damage will limit the functional compensation [19] (relationship pattern b), but the increase in MS-related SC-FC coupling falls somewhere between these two patterns (relationship pattern c). In the dissociation of SC-FC coupling pattern, a normal positive correlation between the SC and FC measures does not exist [31], [54], even a tendency toward a negative correlation (Figure S3). However, to our knowledge, no studies have attempted to link the SC and FC of inter-subregions within the DMN in RRMS patients before this study.

### Relationship between abnormal connectivity indices of inter-subregions and clinical markers of RRMS

This study provided strong evidence in support of a structural disconnection in patients with RRMS. Our study highlighted that altered SC in the long white matter tract [e.g., log (N track) of the PCC/PCUN-left IPL connection, RD values of the MPFC-left IPL connection, FA values of the PCC/PCUN-left mTL connection] correlated with the EDSS, that increases in the RD value of the MPFC-left IPL connection positively correlated with the MFIS and that decreases in the FA value of the PCC/PCUN-right IPL connection negatively correlated with the PASAT. The EDSS is used to quantify and monitor disability changes in MS. A decreased PASAT indicates slowed cognitive processing. The fatigue-related MFIS-5 scale is an abbreviated version used to assess the impact of fatigue on physical, cognitive, and psychosocial functioning. Previous studies have demonstrated that fatigue, EDSS severity and lower PASAT scores in RRMS patient are correlated with the white matter integrity [55], [56]. In this study, we determined that structural damage to the inter-subregions within DMN was associated with clinical markers.

Another highlight of our study was that SC abnormalities of the inter-subregions within the DMN in RRMS patients were significantly correlated with brain atrophy, rather than the total white matter lesion load. Lesion volume is considered to have little correlation with clinical scales when compared with atrophy [38], and our result conformed this opinion.

Furthermore, the FC value of the PCC/PCUN-right mTL was negatively correlated with the PASAT score. Previous studies have demonstrated that the clinical severity of RRMS is inversely correlated with functional connectivity within the DMN [9], [23]. In this study, increases in the FC value of the PCC/PCUN-right mTL were also negatively associated with myelin loss (higher MD values). This association likely occurred because structural damage limits the functional compensation, although functional reorganization was ineffective in the PCC/PCUN-right mTL connection.

### Study Limitations

Importantly, large ROIs could blur the results of functional connectivity analyses. In this study, we defined ROIs that comprised more than one brain area based only on the union of the DMN maps from the RRMS and healthy control groups and without the use of anatomical information or prior seed coordinates from the literature. The selection of the DMN subregions remains controversial in SC-FC coupling studies. Another limitation to this study could be that the DTI probabilistic tractography analyses were restricted to the pair-wise connections. Additionally, there are also a few limitations to probabilistic tractography including the possibly highly reproducible topology of the reconstructed pathways [31], [43] and lack of a statistical consensus on the probabilistic tractography threshold [44]. Moreover, it was demonstrated that robust FC was present between regions not linked by cortico-cortical fiber projections. Limited assessment of cognitive function was implemented in this study. Finally, an explorative study of the altered SC and FC of inter-subregions is warranted in the future to confirm or supplement the findings of this study.

## Conclusions

In the present study, we observed that compared with control subjects, patients with RRMS had significantly reduced structural connections and increased functional connectivity of pair-wise subregions over long distances in the DMN. Specifically, two significant correlations between the FC and SC measures were found in the inter-subregions within the DMN. In addition, relationships between MS-related structural abnormalities and clinical markers were detected more often than were those between functional abnormalities and clinical markers, suggesting that the long-distance connection provided by the white matter is the stable foundation for the function of the entire network. This study, linking the increased inter-subregion functional coherence and decreased SC (long white matter damage) in pair-wise DMN subregions, provides new insights into the understanding of the functional reorganization in RRMS.

## Supporting Information

Supporting Information S1 **Measurement procedures for TWMLL and BPF in the RRMS patients.**
(DOC)

Figure S1 **The mean WM lesion probability distribution map is depicted in colour (see bar on right side) and overlaid on the MNI T_1_ template in the MNI space.**
(TIF)

Figure S2 **Example of the long white matter fibers of pair-wise subregions within the default-mode network was detected in one control subject.** In this figure, the number indicates the subject of detected fibers by probabilistic tractography in the two groups. (PCC/PCUN = posterior cingulate cortex/precuneus, IPL = inferior parietal lobule, mTL = medial temporal lobe, MPFC = medial prefrontal cortex, HC = healthy control, RRMS = relapsing remitting multiple sclerosis. Same abbreviated for all figure and tables).
(TIF)

Figure S3 **An example of the relationship between functional connectivity and structural connectivity measures in others threshold (0.05, 0.10, 0.15, 0.20, 0.25, and 0.30) of probabilistic tractography.**
(TIF)

Table S1 **The union of default mode network subregions from healthy control subjects and RRMS patients (P<0.05, false discovery rate corrected).**
(DOC)

Table S2 **Measures of the structural connectivity (SC) and functional connectivity (FC) of pair-wise default mode subregions between two groups (mean ± standard deviation).**
(DOC)

Table S3 **Comparison of the structural connectivity (SC) and functional connectivity (FC) of pair-wise default mode subregions between two groups [one-way repeated-measure analysis of variance, F values (P values)].**
(DOC)
