# Supplementary material for: Altered Inter-Subregion Connectivity of the Default Mode Network in Relapsing Remitting Multiple Sclerosis: A Functional and Structural Connectivity Study
Source: PLoS One. 2014 Jul 7;9(7):e101198. doi: 10.1371/journal.pone.0101198 (PMC4085052; doi:10.1371/journal.pone.0101198)
Supplement: Supporting Information S1 — Measurement procedures for TWMLL and BPF in the RRMS patients. (DOC) [file pone.0101198.s001.doc]

**Supporting Information for "Altered Inter-subregion Connectivity of the Default Mode Network in Relapsing Remitting Multiple Sclerosis: a Functional and Structural Connectivity Study"**

**Measurement of Total** **White Matter Lesion Loads**

The procedure for the measurement of white matter lesion load in Relapsing Remitting Multiple Sclerosis (RRMS) patients has been previously described . Briefly, the individual *T*_1_-weighted images were first coregistered with the *T*_2_-weighted images using a linear transformation. Next, the transformed *T*_1_-weighted images were normalized to the *T*_1_ template in the Montreal Neurological Institute (MNI) space using a nonlinear transformation. Finally, the transformation information was applied to the lesion masks. This procedure yielded the relative Total White Matter Lesion Loads (TWMLL) for each patient after the removal of the head size effect by normalization. The lesions were re-measured on two separate occasions (at least three months apart) in the patients, and the inter-rater reliability was 94.5%. We generated a distribution map of each lesion by simple superimposition (Figure S1).

**Voxel-based morphometry (VBM) Analysis**

The theory and algorithm of VBM are well-documented by Ashburner and Friston . They used a fully automated whole-brain technique for characterizing regional volume and tissue “concentration” differences in structural MRIs.

3D *T*_1_-weighted images were analyzed with Statistical Parametric Mapping (SPM8; Wellcome Department of Imaging Neurosciences, London, UK; http://www.fil.ion.ucl.ac.uk/spm) running on Matlab (Mathworks, Natick, MA). The first step was generation of a customized template and prior probability maps (specific for gray matter [GM], white matter [WM] and cerebrospinal fluid [CSF]), which were obtained by normalizing the images of all the study subjects in the stereotactic standardized space from the Montreal Neurologic Institute. Next, the original images were spatially normalized to the same stereotactic space (i.e., the customized template) through affine and nonlinear transformations, medium regularization and no masking. The normalized images were partitioned into GM, WM and CSF using the customized prior probability maps. An automated brain extraction tool was used to remove the voxels of nonbrain tissue. Finally, MR imaging random field model-based segmentation was used to segment the brain image into different tissue types, including partial volume models, resulting in a normalized volume of total brain, GM and WM as outputs. In addition, the brain parenchymal fraction (BPF), which is the ratio of brain parenchymal volume to the intracranial volume, may be calculated.

**References**

1. Shu N, Liu Y, Li K, Duan Y, Wang J, et al. (2011) Diffusion tensor tractography reveals disrupted topological efficiency in white matter structural networks in multiple sclerosis. Cereb Cortex 21: 2565-2577.

2. Charil A, Dagher A, Lerch JP, Zijdenbos AP, Worsley KJ, et al. (2007) Focal cortical atrophy in multiple sclerosis: relation to lesion load and disability. Neuroimage 34: 509-517.

3. Ashburner J, Friston KJ (2000) Voxel-based morphometry--the methods. Neuroimage 11: 805-821.
