# Supplementary figures and images for: Altered Inter-Subregion Connectivity of the Default Mode Network in Relapsing Remitting Multiple Sclerosis: A Functional and Structural Connectivity Study
Source: PLoS One. 2014 Jul 7;9(7):e101198. doi: 10.1371/journal.pone.0101198 (PMC4085052; doi:10.1371/journal.pone.0101198)

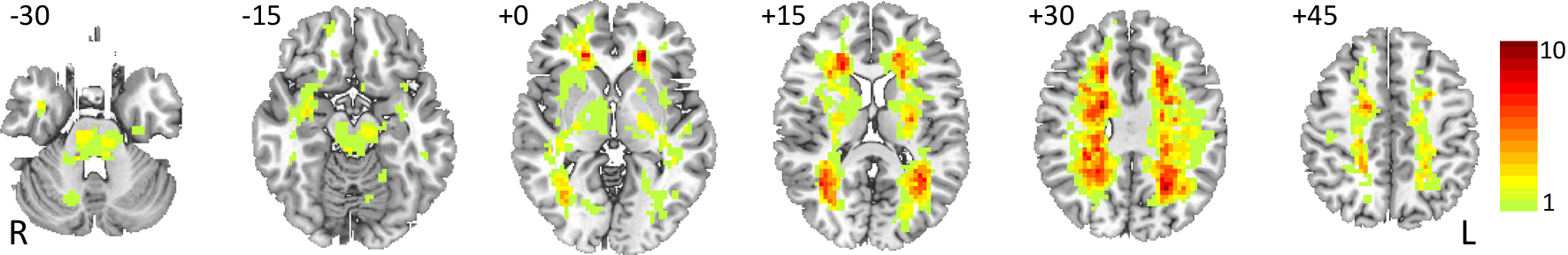

Supplement: Figure S1 — The mean WM lesion probability distribution map is depicted in colour (see bar on right side) and overlaid on the MNI T1 template in the MNI space. (TIF) [file pone.0101198.s002.tif]

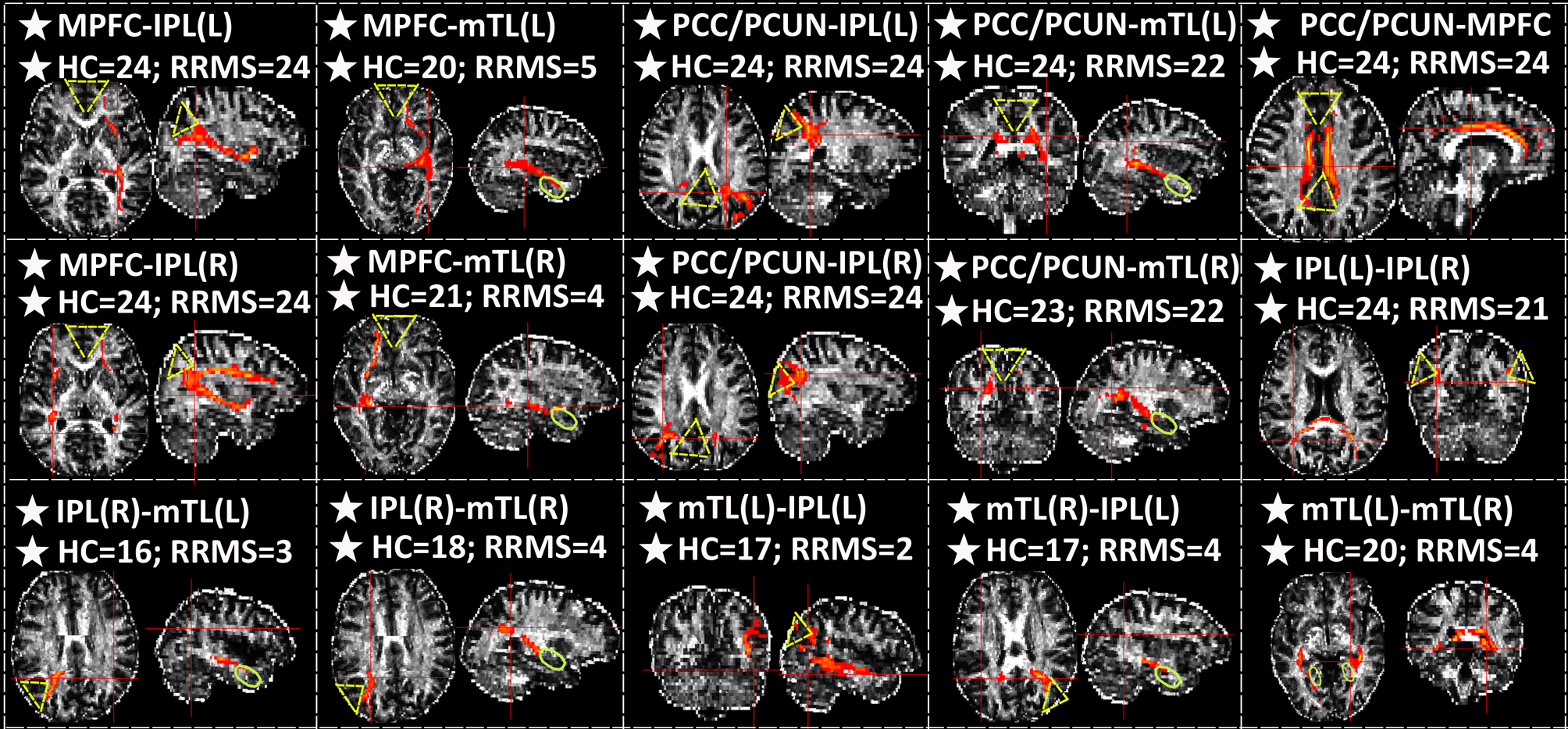

Supplement: Figure S2 — Example of the long white matter fibers of pair-wise subregions within the default-mode network was detected in one control subject. In this figure, the number indicates the subject of detected fibers by probabilistic tractography in the two groups. (PCC/PCUN = posterior cingulate cortex/precuneus, IPL = inferior parietal lobule, mTL = medial temporal lobe, MPFC = medial prefrontal cortex, HC = healthy control, RRMS = relapsing remitting multiple sclerosis. Same abbreviated for all figure and tables). (TIF) [file pone.0101198.s003.tif]

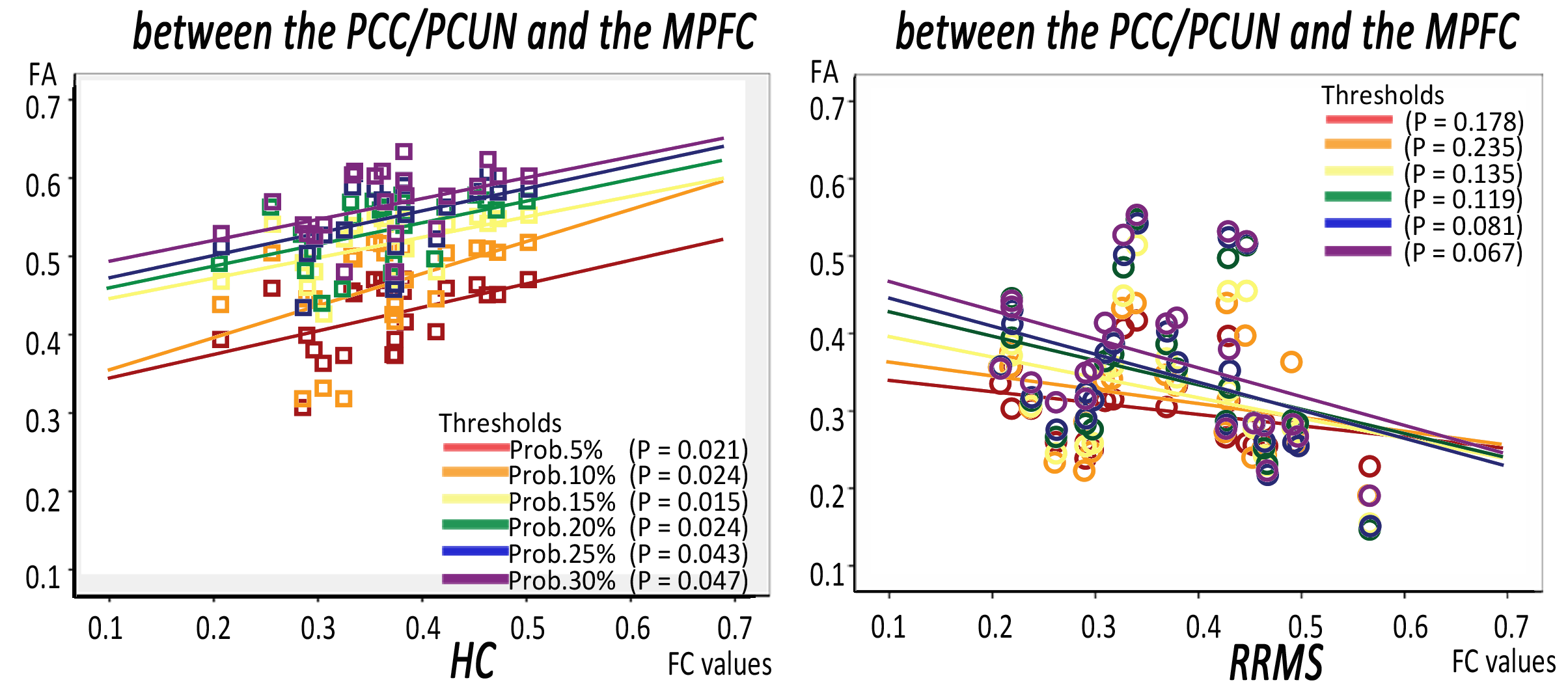

Supplement: Figure S3 — An example of the relationship between functional connectivity and structural connectivity measures in others threshold (0.05, 0.10, 0.15, 0.20, 0.25, and 0.30) of probabilistic tractography. (TIF) [file pone.0101198.s004.tif]
