# Supplementary material for: Altered Inter-Subregion Connectivity of the Default Mode Network in Relapsing Remitting Multiple Sclerosis: A Functional and Structural Connectivity Study
Source: PLoS One. 2014 Jul 7;9(7):e101198. doi: 10.1371/journal.pone.0101198 (PMC4085052; doi:10.1371/journal.pone.0101198)
Supplement: Table S1 — The union of default mode network subregions from healthy control subjects and RRMS patients (P<0.05, false discovery rate corrected). (DOC) [file pone.0101198.s005.doc]

***Table S1***

*The union of default mode network subregions* *from healthy control subjects and RRMS patients (P < 0.05, false discovery rate corrected)*

| Anatomical region | Brodmann’s area | Size (mm^3^) |
| --- | --- | --- |
| PCC/PCUN | 23,27,30 | 52434 |
| MPFC | 8,9,10,11 | 71469 |
| Left IPL | 39,40 | 11286 |
| Right IPL | 39,40 | 10287 |
| Left mTL | 20,30,35 | 2673 |
| Right mTL | 20,30,35 | 2025 |
