# Supplementary material for: Altered Inter-Subregion Connectivity of the Default Mode Network in Relapsing Remitting Multiple Sclerosis: A Functional and Structural Connectivity Study
Source: PLoS One. 2014 Jul 7;9(7):e101198. doi: 10.1371/journal.pone.0101198 (PMC4085052; doi:10.1371/journal.pone.0101198)
Supplement: Table S2 — Measures of the structural connectivity (SC) and functional connectivity (FC) of pair-wise default mode subregions between two groups (mean ± standard deviation). (DOC) [file pone.0101198.s006.doc]

***Table S2*** *Measures of the structural connectivity (SC) and functional connectivity (FC) of pair-wise default mode subregions between two groups (mean ± standard deviation)*

|  | | MPFC- PCC/PCUN | MPFC - Left IPL | MPFC - Right IPL | MPFC - Left mTL | MPFC - right mTL | PCC/PCUN - Left IPL | PCC/PCUN - Right IPL | PCC/PCUN - Left mTL | PCC/PCUN - Right mTL | | Right IPL - Left IPL© | | Left IPL - Left mTL | Left IPL - Right mTL | Right IPL - Left mTL | Right IPL - Right mTL | | Right mTL - Right mTL |
| --- | --- | --- | --- | --- | --- | --- | --- | --- | --- | --- | --- | --- | --- | --- | --- | --- | --- | --- | --- |
| Correlation coefficient (r- values) | RRMS | 0.41±0.11 | 0.47±0.15 | 0.31±0.13 | 0.20±0.13 | 0.14±0.09 | 0.42±0.14 | 0.49±0.20 | 0.30±0.13 | | 0.25±0.17 | 0.45±0.18 | 0.26±0.07 | | 0.21±0.15 | 0.13±0.11 | 0.26±0.08 | 0.24±0.17 | |
|  | HC | 0.35±0.11 | 0.35±0.12 | 0.23±0.14 | 0.22±0.14 | 0.15±0.06 | 0.43±0.16 | 0.44±0.15 | 0.20±0.06 | | 0.11±0.05 | 0.51±0.13 | 0.20±0.03 | | 0.05±0.03 | 0.15±0.10 | 0.07±0.13 | 0.11±0.04 | |
| Detectable of tractography (n) | RRMS | 24 | 24 | 24 | 5 | 4 | 24 | 24 | 22 | | 22 | 21 | 2 | | 4 | 3 | 4 | 2 | |
|  | HC | 24 | 24 | 24 | 20 | 21 | 24 | 24 | 24 | | 23 | 24 | 17 | | 17 | 16 | 18 | 20 | |
| mean track count (logN) | RRMS | 5.21±0.37 | 3.16±0.81 | 2.92±0.91 | 2.04±0.91 | 2.33±0.18 | 4.66±0.71 | 4.61±0.33 | 2.83±0.41 | | 2.84±0.58 | 2.26±1.19 | 3.30±0.14 | | 2.87±0.53 | 2.98±0.23 | 3.00±0.34 | 3.20±0.43 | |
|  | HC | 5.69±0.06 | 3.31±0.44 | 3.24±0.62 | 4.10±0.49 | 3.74±0.49 | 4.69±0.31 | 4.90±0.35 | 4.77±0.54 | | 4.90±0.23 | 2.52±0.92 | 4.55±0.48 | | 2.91±0.95 | 2.74±0.55 | 4.58±0.28 | 4.43±0.45 | |
| Volumes of tract(×10^3^ mm^3^) | RRMS | 2.26±0.55 | 3.11±1.25 | 2.17±1.05 | 1.28±0.95 | 1.21±1.18 | 2.69±1.22 | 3.17±1.59 | 2.92±1.53 | | 2.89±1.37 | 3.51±1.05 | 0.76±0.32 | | 1.59±0.86 | 0.90±0.23 | 1.21±0.35 | 1.45±0.97 | |
|  | HC | 4.55±0.98 | 3.47±1.15 | 3.88±1.71 | 3.53±0. 96 | 3.21±0.95 | 2.38±0.72 | 3.08±0.95 | 3.23±1.07 | | 3.09±0.77 | 3.56±2.38 | 1.63±0.43 | | 3.07±0.15 | 3.14±0.53 | 1.62±0.09 | 3.22±0.78 | |
| FA values on track | RRMS | 0.311±0.094 | 0.342±0.054 | 0.342±0.025 | 0.365±0.083 | 0.290±0.056 | 0.290±0.083 | 0.295±0.036 | 0.375±0.050 | | 0.335±0.063 | 0.3160.102 | 0.322±0.028 | | 0.331±0.087 | 0.342±0.046 | 0.308±0.059 | 0.384±0.062 | |
|  | HC | 0.541±0.096 | 0.463±0.022 | 0.453±0.025 | 0.483±0.032 | 0.469±0.036 | 0.474±0.053 | 0.538±0.058 | 0.548±0.036 | | 0.568±0.025 | 0.549±0.099 | 0.462±0.062 | | 0.555±0.049 | 0.544±0.032 | 0.493±0.032 | 0.495±0.049 | |
| MD values on track | RRMS | 0.965±0.019 | 0.994±0.146 | 0.994±0.103 | 0.967±0.089 | 0.691±0.065 | 0.949±0.123 | 0.924±0.116 | 1.041±0.943 | | 0.96±0.113 | 0.928±0.819 | 1.265±0.111 | | 1.045±0.659 | 1.186±0.978 | 1.113±0.056 | 1.26±0.132 | |
|  | HC | 0.691±0.038 | 0.747±0.045 | 0.765±0.047 | 0.733±0.037 | 0.738±0.037 | 0.725±0.029 | 0.716±0.024 | 0.79±0.046 | | 0.801±0.062 | 0.747±0.062 | 0.708±0.035 | | 0.841±0.014 | 0.866±0.066 | 0.699±0.012 | 1.112±0.073 | |
| AD values on track | RRMS | 1.259±0.172 | 1.318±0.142 | 1.318±0.234 | 1.319±0.136 | 1.09±0.145 | 1.216±0.119 | 1.176±0.351 | 1.418±0.113 | | 1.439±0.095 | 1.215±0.109 | 1.358±0.116 | | 1.424±0.127 | 1.186±0.131 | 1.322±0.153 | 1.737±0.255 | |
|  | HC | 1.117±0.070 | 1.147±0.059 | 1.158±0.078 | 1.154±0.057 | 1.147±0.056 | 1.143±0.098 | 1.193±0.068 | 1.295±0.064 | | 1.33±0.066 | 1.264±0.061 | 1.09±0.054 | | 1.378±0.068 | 1.405±0.083 | 1.108±0.054 | 1.676±0.083 | |
| RD values on track | RRMS | 0.790±0.201 | 0.822±0.148 | 0.822±0.230 | 0.829±0.103 | 0.441±0.098 | 0.815±0.135 | 0.814±0.135 | 0.729±0.201 | | 0.753±0.127 | 0.792±0.131 | 0.585±0.065 | | 0.824±0.107 | 0.652±0.067 | 0.671±0.131 | 0.856±0.165 | |
|  | HC | 0.485±0.084 | 0.548±0.040 | 0.571±0.068 | 0.471±0.064 | 0.479±0.060 | 0.507±0.058 | 0.474±0.033 | 0.479±0.034 | | 0.482±0.026 | 0.485±0.027 | 0.461±0.032 | | 0.505±0.025 | 0.532±0.028 | 0.441±0.024 | 0.757±0.045 | |

*Note: For each connection, differences at the significant level of P < 0.05 Bonferroni corrected, P < 0.01 Bonferroni corrected and P < 0.001 Bonferroni corrected were marked with *, ** and ***, respectively. Three diffusivity measurements (reported in units of**×**10-3mm2/s). AD = axial diffusivity, FA= fractional anisotropy, HC = healthy control, IPL = inferior parietal lobule, MD = mean diffusivity, mTL = medial temporal lobes, MPFC = medial prefrontal cortex, PCC/PCUN = posterior cingulate cortex/precuneus, RD = radial diffusivity. (same for all tables)*
