# Supplementary material for: Altered Inter-Subregion Connectivity of the Default Mode Network in Relapsing Remitting Multiple Sclerosis: A Functional and Structural Connectivity Study
Source: PLoS One. 2014 Jul 7;9(7):e101198. doi: 10.1371/journal.pone.0101198 (PMC4085052; doi:10.1371/journal.pone.0101198)
Supplement: Table S3 — Comparison of the structural connectivity (SC) and functional connectivity (FC) of pair-wise default mode subregions between two groups [one-way repeated-measure analysis of variance, F values (P values)]. (DOC) [file pone.0101198.s007.doc]

***Table S3*** ***Comparison of the structural connectivity (SC) and functional connectivity (FC) of pair-wise default mode subregions between two groups [one-way repeated-measure analysis of variance, F values (P values)]***

|  | MPFC - PCC/PCUN | MPFC - Left IPL | MPFC - Right IPL | MPFC - Left mTL | MPFC - right mTL | PCC/PCUN - Left IPL | PCC/PCUN - Right IPL | PCC/PCUN - Left mTL | PCC/PCUN - Right mTL | Right IPL - Left IPL | Left IPL - Left mTL | Left IPL - Right mTL | Right IPL - Left mTL | Right IPL - Right mTL | Right mTL - Right mTL |
| --- | --- | --- | --- | --- | --- | --- | --- | --- | --- | --- | --- | --- | --- | --- | --- |
| Correlation coefficient | 2.637  (0.119) | 8.555  (0.005) | 3.606  (0.064) | 0.430  (0.515) | 0.280  (0.599) | 0.079  (0.779) | 1.089  (0.302) | **10.85**  **(0.002)** | **13.58**  **(0.001)** | 1.221  (0.275) | **11.71**  **(0.001)** | **12.99**  **(0.0007)** | 0.313  (0.578) | **14.0**  **(0.0001)** | **13.58**  **(0.001)** |
| Detectable of tractography | na/na | na/na | na/na | **19.57**  **(0.000)** | **16.48**  **(0.000)** | na/na | na/na | 2.09  (0.155) | 0.343  (0.561) | 3.286  (0.076) | **13.748**  **(0.000)** | **19.533**  **(0.000)** | **12.351**  **(0.000)** | **13.979**  **(0.000)** | **16.09**  **(0.000)** |
| mean track count(logN) | **18.73**  **(0.000)** | 0.631  (0.431) | 2.019  (0.162) |  |  | 0.023  (0.880) | **8.203**  **(0.004)** | **12.8**  **(0.000)** | **14.8**  **(0.000)** | 0.640  (0.428) |  |  |  |  |  |
| Volumes of tract | **9.783**  **(0.003)** | 0.642  (0.423) | **17.31**  **(0.000)** |  |  | 1.123  (0.295) | 0.064  (0.801) | **4.52**  **(0.042)** | **4.47**  **(0.047)** | 0.008  (0.930) | - | - | - | - | - |
| FA values on track | **18.19**  **(0.000)** | **10.05**  **(0.000)** | **16.102**  **(0.000)** |  |  | **13.13**  **(0.000)** | **13.93**  **(0.000)** | **15.701**  **(0.000)** | **16.106**  **(0.000)** | **12.717**  **(0.000)** |  |  |  |  |  |
| MD values on track | **14.32**  **(0.000)** | **13.00**  **(0.000)** | **18.241**  **(0.000)** |  |  | **14.96**  **(0.000)** | **13.77**  **(0.000)** | **17.56**  **(0.000)** | **12.05**  **(0.002)** | **17.11**  **(0.000)** |  |  |  |  |  |
| AD values on track | **13.897**  **(0.001)** | **12.409**  **(0.000)** | **10.145**  **(0.003)** |  |  | 2.312  (0.18) | 0.338  (0.564) | **4.46**  **(0.046)** | **5.13**  **(0.039)** | **8.683**  **(0.004)** |  |  |  |  |  |
| RD values on track | **14.70**  **(0.000)** | **15.02**  **(0.000)** | **27.63**  **(0.000)** |  |  | **14.04**  **(0.000)** | **18.7**  **(0.000)** | **5.46**  **(0.037)** | **4.34**  **(0.047)** | **12.9**  **(0.000)** |  |  |  |  |  |
